# Supplementary figures and images for: The Effects of Alcohol Drinking on Oral Microbiota in the Chinese Population
Source: Int J Environ Res Public Health. 2022 May 8;19(9):5729. doi: 10.3390/ijerph19095729 (PMC9103016; doi:10.3390/ijerph19095729)

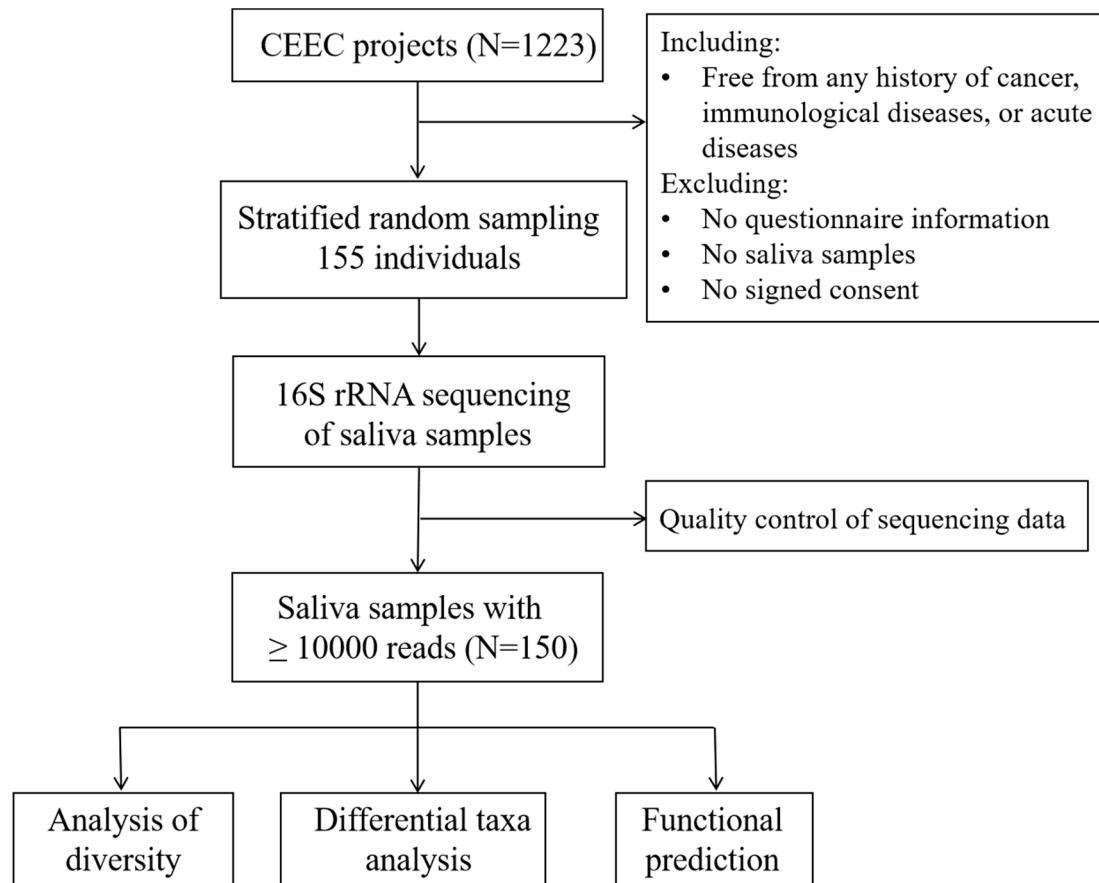

**Supplementary Figure S1: The overflow chart of this study.**

Supplement: Supplementary file 1 [file ijerph-19-05729-s001.zip › Supplementary figure.pdf]
